# Population Genomics Reveals the Underlying Structure of the Small Pelagic European Sardine and Suggests Low Connectivity within Macaronesia

**DOI:** 10.3390/genes15020170

**Published:** 2024-01-27

**Authors:** Rute R. da Fonseca, Paula F. Campos, Alba Rey-Iglesia, Gustavo V. Barroso, Lucie A. Bergeron, Manuel Nande, Fernando Tuya, Sami Abidli, Montse Pérez, Isabel Riveiro, Pablo Carrera, Alba Jurado-Ruzafa, M. Teresa G. Santamaría, Rui Faria, André M. Machado, Miguel M. Fonseca, Elsa Froufe, L. Filipe C. Castro

**Affiliations:** 1 Center for Global Mountain Biodiversity, GLOBE Institute, University of Copenhagen, Universitetsparken 15, 2100 Copenhagen, Denmark; 2 The Bioinformatics Centre, Department of Biology, University of Copenhagen, 2200 Copenhagen, Denmark; campos.f.paula@gmail.com; 3 CIIMAR—Interdisciplinary Centre of Marine and Environmental Research, University of Porto, 4050-123 Porto, Portugal; mnande@ciimar.up.pt (M.N.); r.macieiradefaria@sheffield.ac.uk (R.F.); andre.machado@ciimar.up.pt (A.M.M.); mig.m.fonseca@gmail.com (M.M.F.); elsafroufe@gmail.com (E.F.); 4 Centre for GeoGenetics, Natural History Museum Denmark, University of Copenhagen, Østervoldgade 5-7, 1350 Copenhagen, Denmark; ardelaiglesia@sund.ku.dk; 5 Department of Ecology and Evolutionary Biology, University of California, Los Angeles, CA 90095, USA; gvbarroso@gmail.com; 6 Section for Ecology and Evolution, University of Copenhagen, 2100 Copenhagen, Denmark; lucie.a.bergeron@gmail.com; 7 Grupo en Biodiversidad y Conservación, IU-ECOAQUA, Universidad de Las Palmas de Gran Canaria, 35017 Las Palmas, Spain; ftuya@yahoo.es; 8 Laboratory of Environment Bio-Monitoring, Faculty of Sciences of Bizerte, University of Carthage, Bizerte 7021, Tunisia; abidli_sami@yahoo.fr; 9 Centro Oceanográfico de Vigo, Instituto Español de Oceanografía, IEO-CSIC, 36390 Vigo, Spain; montse.perez@ieo.csic.es (M.P.); isabel.riveiro@ieo.csic.es (I.R.); pablo.carrera@ieo.csic.es (P.C.); 10 Centro Oceanográfico de Canarias, Instituto Español de Oceanografía, IEO-CSIC, 38180 Santa Cruz de Tenerife, Spain; alba.jurado@ieo.csic.es (A.J.-R.); mtgarsantamaria@gmail.com (M.T.G.S.); 11 Department of Biology, Faculty of Sciences, University of Porto, 4169-007 Porto, Portugal

**Keywords:** European sardine, low coverage sequencing, population structure, marine fish

## Abstract

The European sardine (*Sardina pilchardus*, Walbaum 1792) is indisputably a commercially important species. Previous studies using uneven sampling or a limited number of makers have presented sometimes conflicting evidence of the genetic structure of *S. pilchardus* populations. Here, we show that whole genome data from 108 individuals from 16 sampling areas across 5000 km of the species’ distribution range (from the Eastern Mediterranean to the archipelago of Azores) support at least three genetic clusters. One includes individuals from Azores and Madeira, with evidence of substructure separating these two archipelagos in the Atlantic. Another cluster broadly corresponds to the center of the distribution, including the sampling sites around Iberia, separated by the Almeria–Oran front from the third cluster that includes all of the Mediterranean samples, except those from the Alboran Sea. Individuals from the Canary Islands appear to belong to the Mediterranean cluster. This suggests at least two important geographical barriers to gene flow, even though these do not seem complete, with many individuals from around Iberia and the Mediterranean showing some patterns compatible with admixture with other genetic clusters. Genomic regions corresponding to the top outliers of genetic differentiation are located in areas of low recombination indicative that genetic architecture also has a role in shaping population structure. These regions include genes related to otolith formation, a calcium carbonate structure in the inner ear previously used to distinguish *S. pilchardus* populations. Our results provide a baseline for further characterization of physical and genetic barriers that divide European sardine populations, and information for transnational stock management of this highly exploited species towards sustainable fisheries.

## 1. Background

Population structuring in the absence of obvious physical barriers has puzzled biologists for centuries. A lack of or reduced genetic structure is expected in an oceanic environment. This is because most marine organisms are capable of exchanging migrants across large distances in long larval pelagic phases, plus they have high fecundity, large population sizes and adult migratory behavior [1,2]. Yet, many studies have shown that several species have higher spatial genetic differentiation than expected considering their high dispersal potential [3,4]. In the case of marine fish, structure can range from a lack of differentiation between oceans to significant structure within an ocean basin [2], challenging the simple concept of “open seas” and the assumption of high connectivity in marine environments [5]. Assessing the existence of population structure in marine species capable of long-distance dispersal is essential to identify the various factors involved in population differentiation and diversification in the absence of complete physical barriers [6]. This is especially relevant for conservation efforts, including stock management of commercially important species [1,2,7].

The Mediterranean Sea and the contiguous Northeastern Atlantic Ocean have been the focus of several phylogeographic and population genetic studies on marine fish (e.g., [8,9]). The Almeria–Oran Front, a well-defined oceanographic break situated east of the Strait of Gibraltar, has been suggested to be responsible for hindering gene flow between Mediterranean and Atlantic fish populations of many fish species but it is far from being an universal barrier [8]. The less studied Macaronesian region, a group of archipelagos (Azores, Madeira and Canaries) separated from the Euro-African mainland by c. 100–1900 km, has also been the target of several phylogeographic studies (e.g., [10,11]). This region is characterized by the presence of several oceanographic currents, e.g., the North Atlantic Current, the Azores Current and the Canary Current [12], that together with the apparent lack of physical barriers can strengthen the potential for gene flow. Therefore, it is not surprising that several studies have reported low population genetic differentiation within the Macaronesian region for different taxa [1], including fishes [13,14]. Species distributed across these regions can thus inform us about the existence of cryptic substructure and possible barriers to gene flow among populations.

One of the most important pelagic fish resources in Atlantic waters is the European sardine, *Sardina pilchardus* Walbaum, 1792. This species has an enormous economic value, especially in Southern Europe and Morocco [15], where it is a main target of the purse-seine fleets in Portugal and Spain, representing a major source of income for local economies [16]. Recently reported low biomass levels [17] led to a recommendation for reduced fishing in Southern Europe, with great economic impact. It also prompted us to reevaluate the current population structure of *S. pilchardus*, aiming at the ongoing discussion on how genetic information can contribute to stock delineation for management purposes [7].

The European sardine has a broad distribution from the Eastern Mediterranean to the North-East Atlantic, including the Azores, Madeira and the Canary Islands’ Archipelagos, and is found along the African coast down to Senegal [18]. Like other marine pelagic fish, *S. pilchardus* shows schooling and migratory behavior and high dispersal capabilities, both at the larval and adult stages. In agreement, low levels of genetic differentiation were detected across the species distribution using allozymes [19,20,21,22], mitochondrial DNA (mtDNA) [23,24], and microsatellites [10,25]. Nevertheless, phenotypic variation in gill raker counts and head length [18,26], as well as mitochondrial haplotype frequency differences [23] led to the proposal of two subspecies: *S. pilchardus pilchardus* (North Sea to southern Portugal) and *S. pilchardus sardina* (Mediterranean Sea and northwest African coast). Accordingly, otolith shapes differ between Atlantic and Mediterranean sardines [27,28], and further suggest a subdivision between the Northern Mediterranean and the Alboran–Algero-Provençal basin [27,29]. A study using 15 allozymes supports the latter [30], but, unlike the otolith shapes, these markers suggest discontinuity caused by the Almeria–Oran front. When considering a large fraction of the European sardine Atlantic range, allozymes and microsatellites suggest that Madeira and Azores form a significantly differentiated group [10]. This mosaic of regional population structure built by several independent studies has been mostly justified by geographical barriers that potentially hinder gene flow, expected to be high for the abundant and mobile *S. pilchardus*. The phenotypic differences between groups might also have arisen from retention of adaptive phenotypes, as population structure in the Mediterranean was found to be associated with environmental variables [31]. This prompted us to raise questions about the contributions of genomic architecture to the basis of the observed present-day population structure.

In this study, we produced a European sardine genomic data set consisting of whole genome nuclear data and complete mitochondrial genomes for 88 individuals that were analyzed together with data from 20 sardine individuals from a previous study [32], in a total of 108 samples from 15 locations across 5000 km of the species distribution range. This enabled us to investigate previously suggested barriers to gene flow, map the major genetic clusters that characterize *S. pilchardus* in a large part of its distribution, compare markers with different modes of inheritance, and also reveal the first insights into the genomic barriers contributing to the observed population structure.

## 2. Materials and Methods

### 2.1. Sample Collection, DNA Extraction and Sequencing

**Figure 1:**
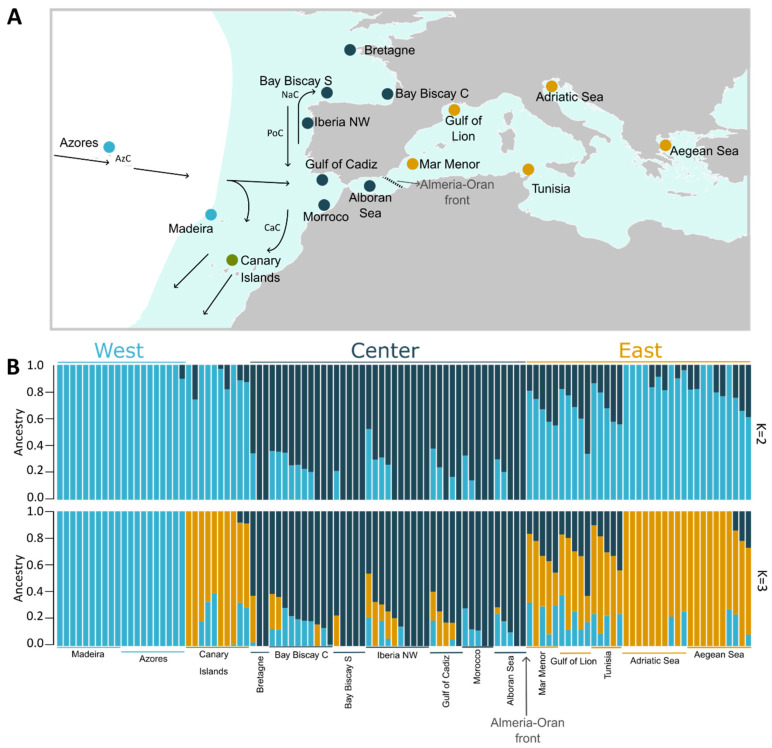
(**A**) Sampling sites across the species distribution (blue area, adapted from FAO). The color of each circle represents the most frequent genetic cluster for K = 3 (light blue: West; dark blue: Center; yellow: East; the Canary Isalnds are depicted in green). Surface currents are represented by arrows: Azores Current (AzC); Canary Current (CaC); Portugal Current (PoC); and Navidad Current (NaC). The Almeria–Oran Front (AO) is shown as a dashed line. (**B**) Population structure plot showing the ancestry of each individual (vertical bar) to two (above) and three (below) genetic clusters.

Samples originate from 15 different geographical locations encompassing a large part of the species’ current distribution range (Figure 1A, Table 1). Samples from the Alboran Sea and from Central and Western Bay of Biscay (n = 15 individuals) were collected during the PELACUS oceanographic surveys. The remaining 73 specimens were collected at local markets from ten distinct geographic locations (Table S1). Additionally, sequence data for samples from the Gulf of Cadiz, Mar Menor, the Gulf of Lion, and a second set for Central Bay of Biscay, were obtained from Barry et al. (2022) [32].

Total genomic DNA was extracted using Qiagen’s DNeasy Blood & Tissue Kit (Hilden, Germany) according to the manufacturer’s instructions, with the following modifications: prior to elution in 100 µL AE buffer, samples were incubated at 37 °C for 10 min to increase DNA yield. DNA concentration and purity were verified using a Nanodrop Spectrophotometer and a Qubit Fluorometer. A commercial service (Novogene, Guangzhou City, China) produced Truseq Nano DNA libraries and sequenced paired-end reads (150 base pairs (bp)) in a Novaseq6000. To assess the patterns of genetic differentiation of the European sardine, 81 samples were sequenced to 3X sequencing depth (i.e., each position of the genome is covered by 3 reads on average) and 7X to 20X sequencing depth (details in Supplementary File S1: Table S1). Raw data for 20 sardine individuals from Barry et al. [32] were further processed using the same procedure as described in the next section (sequencing depth between 15 and 22X). Table S1 indicates the assignment of samples to the different subsets considered for further analysis.

### 2.2. Assembly Filtering and Re-Sequencing Data Pre-Processing

Individual contigs in the reference genome (GenBank assembly accession: GCA_900499035.1) of *S. pilchardus* [33] matching mitochondrial DNA (unique assignment to mitochondrial DNA without nuclear genome flanking regions) were identified via BLAST (version 2.6.0+) [34] using the mitochondrial genome (mtDNA) assembled by Machado et al. [35] as a query. Matching contigs were removed from the assembly file and replaced by the mtDNA of Machado et al. (2018) [35] to enable the extraction of individual mtDNA sequences from all individuals after mapping of resequencing data.

Regions of low complexity in the reference genome of *S. pilchardus* [33] were detected with GenMap (version v1.3.0) [36] using a *k*-mer of 100 bp. We calculated the normalized depth per scaffold using the sequencing depth of scaffold 1 as a reference to identify potential misassemblies (e.g., unmerged haploid scaffolds or collapsed repeats regions). Regions of mappability below 1 (meaning that more than one 100 bp kmer matched the region, indicating duplications) or identified as repeats in all the other scaffolds, and sites with data missing in more than 25% of the individuals were excluded from all subsequent analyses.

Raw Illumina reads for all 108 samples were first processed with Trimmomatic (version 0.36) [37] for removal of adapter sequences, trimming bases with quality <20 and discarding reads with length <80. Clean reads were mapped to the genome assembly using bwa-mem version: 0.7.17-r1188 [38], and samtools version: 1.7 [39] was used to retain reads with mapping quality >25. PCR duplicates were removed with Picard MarkDuplicates (version 1.95; http://broadinstitute.github.io/picard/) and only reads where both pairs were retained were considered for the local realignment around indels with GATK version 3.6–0-g89b7209 [40] and further analyses. The mapping and base quality options -minQ 20 -minMapQ 30 were used in all subsequent analyses with ANGSD [41].

### 2.3. Population Structure

Beagle files with the nuclear genome positions of single nucleotide polymorphisms (SNPs) were produced by ANGSD [41] using the following options: -GL 1 -doGlf 2 -minMaf 0.05 -C 50 -baq 2 -remove_bads 1 -uniqueOnly 1 -SNP_pval 1e−6. Linkage disequilibrium (LD) was estimated as *r*^2^ values for all SNP pairs minimum 500 kbp apart with ngsLD v1.1.1 [42], and an LD decay curve was plotted using 0.05% of all estimated *r*^2^ values. This indicated that a distance threshold of 2000 bp was adequate for linkage pruning. A total of 560,735 SNPs were obtained using all samples (n = 108, Table S1), and the 319,236 SNPs located in putatively neutral regions of the genome (50 kb genomic windows with PBS values below the 90th percentile, details below; Figure S2) were used in all the population genomic analyses. Admixture proportions were estimated by running NGSadmix version 32 [43] for K equal to 2 and 3 with 300 seed values, ensuring convergence (convergence was not reached for K = 4 and above). A principal component analysis (PCA) using the same SNP set was obtained with PCAngsd version 0.1 [44].

The mitochondrial genome (mtDNA) for each individual was obtained as a consensus sequence of the reads mapped to the mtDNA sequence included in the reference genome using the option -doFasta 2 and removing positions with sequencing depth below 10X (-setMinDepth 10) in ANGSD [41]. A haplotype network was reconstructed using mitochondrial SNPs with minor allele frequency >30% (total of 26 SNPs) in POPART [45] with the Median Joining Network algorithm [46].

### 2.4. Population Differentiation

**Figure 2:**
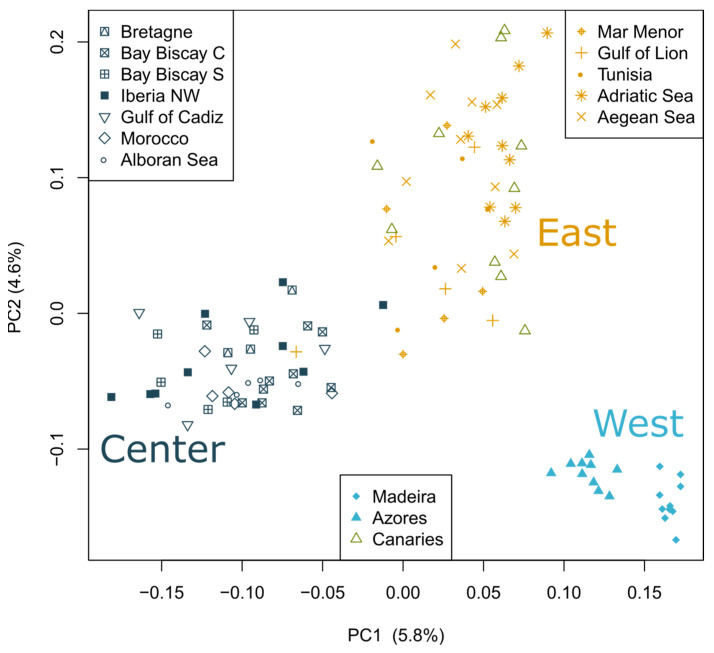
Distribution of individuals based on the first three components of the principal component analysis. Variance explained by each component is shown in parentheses.

**Figure 3:**
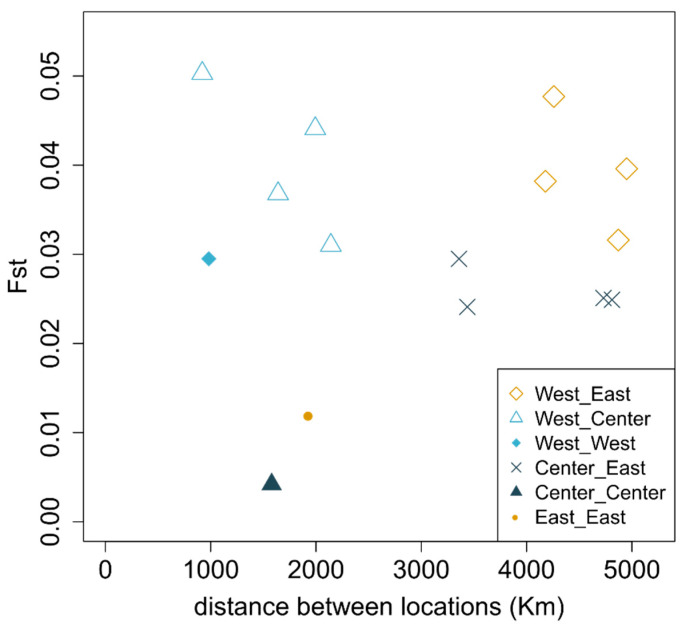
Average pairwise *F_ST_* between populations with minimum of 10 individuals (Table S2) calculated across 50 kb windows located in putatively neutral regions of the genome. The correlation between distance and *F_ST_* is not significant (*Mantel* test; *p*-value = 0.32).

We used methods based on the site frequency spectrum (SFS) [47,48] to obtain the genome-wide fixation index (F_ST_) values in ANGSD [41]. We calculated F_ST_ for the populations containing at least 10 individuals with each geographical region represented by two locations (West: Madeira and Azores; East: Adriatic and Aegean; Center: Bay of Biscay and Gulf of Cádiz/Morocco; Table S2; Figure 2; Figure 3). First, we generated unfolded SAF files (angsd -bam bamList -doSaf 1 -anc ANC -GL 1) and then we estimated the folded SFS for each pair of populations (realSFS safidx1 safidx2 -fold 1). Each joint folded SFS was then used to estimate F_ST_ (-whichFst 1 -fold 1). Table S2 also includes values for the 25th and 75th percentile of the distribution of F_ST_ values calculated per 50 kb windows along the genome. To detect genomic windows of high differentiation in each region, we estimated the population branch statistic [49] for non-overlapping windows of 50 kb in ANGSD using this same approach with three populations with 10 individuals each (Tables S1–S3). The individuals were chosen from those that had 100% assignment to one of the three ancestral populations defined by NGSadmix in preliminary analysis using all genomic positions that passed the filters described above.

Maritime geographical pairwise distances (https://sea-distances.org/, accessed on 2 June 2022) were calculated using the seaport nearest to the sampling location. Average distances were considered for merged populations. A Mantel test implemented in ade4 [50] was used to test the statistical significance of the correlation between the geographic and the genetic distance matrices.

We assessed the gene content of the top outlier PBS windows (outliers in the 99th percentile; Figure S2) for each region by running tblastn [34] (BLAST version 2.6.0+) of the zebrafish proteome (ENSEMBL version GRCz11_pep) against the reference genome using the option “-evalue 0.000001”. Phenotypes associated with each gene were extracted from ENSEMBL using Biomart [51].

### 2.5. Recombination Rate

Variants were called using GATK version 4.0.7.0 [40] for one representative individual per region (Table S1). Briefly, variants were first called for each individual with HaplotypeCaller in BP-RESOLUTION mode; then those GVCF files for each sample were combined into a single one using CombineGVCFs per scaffold of interest, followed by joint genotyping with GenotypeGVCF. The default filter of GATK (--phred-scaled-global-read-mismapping-rate 45 --base-quality-score-threshold 18 --min-base-quality-score 10) was used. Recombination rates for 100 kb non-overlapping windows along the genome were estimated using the iSMC approach from [52]. We fitted an iSMC model with 40-time intervals and five categories of recombination rates to the samples from each population and optimized parameters in composite likelihood fashion [53]. We then obtained recombination landscapes of single-nucleotide resolution by performing posterior decoding in each diploid using the estimated parameters and computed a consensus map for each sample by averaging (for each site) the posterior estimates of rho = 4*Ne*r from all diploids. The final map of 100 kb resolution was obtained by further averaging the single-nucleotide estimates over 100 kb in non-overlapping windows.

## 3. Results

### 3.1. Population Structure

The admixture analysis conducted in NGSadmix showed that European sardines comprise three genetic clusters (Figure 1B). When setting the number of expected clusters to two (k = 2; Figure 1B, top), one of the clusters is prevalent in the Center region, while the other is more frequent in both Western and Eastern regions, as well as the Canary Islands. Individuals showing patterns compatible with admixed ancestry between these two clusters were observed at all sampling sites, except at Madeira. For k = 3 (Figure 1B, bottom), one of these clusters (West–East–Canaries) splits into two: one frequent in the Mediterranean and Canaries and the other in the West (Madeira and Azores). Patterns compatible with admixed ancestry among the three main clusters were observed in individuals from the Central region, Eastern Iberia, South of France, Tunisia and the Aegean Sea. A few individuals from Canaries and Adriatic show some admixed ancestry with the Western cluster. The proportion of individuals with admixed ancestry is lower in populations located in the extremes of the distribution: Madeira, Azores in the West, Aegean and Adriatic Sea in the East, as well as Bretagne and Bay of Biscay S in the central group.

Three separate clusters of populations can also be observed in the principal component analysis (PCA; Figure 2). The first two PCs explained 5.8% and 4.6% of the total variation. Within the three main clusters, individuals from the same sampling location do not cluster together, likely reflecting admixture, as observed in Figure 1B, except for Madeira and Azores, which appear separated in the PC1.

The main clusters observed in the mitochondrial haplotype network combine haplotypes that are not geographically confined to a region, suggestive of gene flow mainly between Center and East (Figure S1), which is consistent with their proximity in PC2 (Figure 2). The West group dominates a centrally branching haplogroup.

### 3.2. Population Differentiation

**Figure 4:**
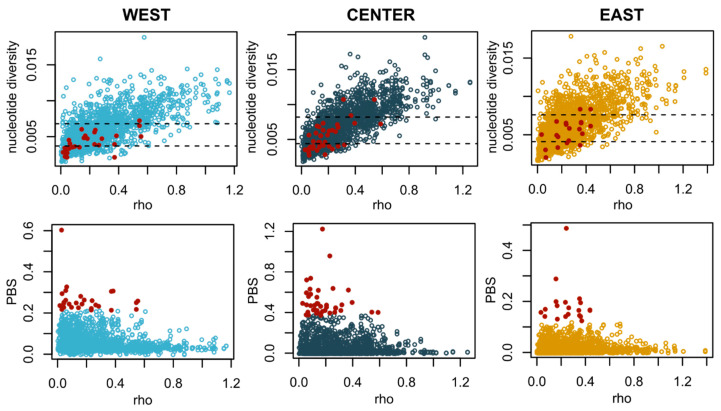
Summary statistics calculated across 50 kb windows located in putatively neutral regions of the genome located in comparison with the top outliers (99th percentile) of the PBS analysis for each region (red dots). **Top**: Nucleotide diversity vs. recombination rate. **Bottom**: PBS vs. recombination rate.

The levels of nucleotide diversity were comparable across regions, although slightly lower for populations in the West (Table S2). In general, we observed lower values of genetic differentiation as measured by F_ST_ for comparisons within regions (distances ranging from 982 to 1943 km show F_ST_ values between 0.004 and 0.03; Figure 3 and Table S3). The highest values of F_ST_ included comparisons with Madeira (West) and sampling locations in the Center region and the Aegean Sea (0.044 < F_ST_ < 0.05). We do not find evidence of isolation by distance across the sampled range (*Mantel* test; *p*-value = 0.32). Areas of the genome that correspond to the top PBS outliers (99th percentile, corresponding to the genomic regions where one population is the most dissimilar to the other two) show lower recombination rates, and areas of very low differentiation are associated with the highest recombination rates (Figure 4). The gene content of the windows with the top individual PBS values per geographical location (Figure S2) includes proteins involved in otolith formation (East), vasculature and organ formation (Center) and blood coagulation (West) (Table S4).

## 4. Discussion

In this study, we present the first analysis of population structure in European sardine across a large part of its distribution range using whole-genome sequencing data. A number of mechanisms have been suggested to explain how population structure can evolve in an environment without any complete physical barrier to gene flow, including local adaptation, habitat discontinuity, different habitat preferences and behavior, sexual selection, oceanographic currents, isolation by distance and limited dispersal capabilities [6,8,54,55,56].

Altogether, the assessment of nuclear genome sequences by means of individual ancestry information, principal component analysis (Figure 1B and Figure 2) and differentiation (F_ST_) among populations from different geographic regions (Figure 3), supports that the European sardine comprises three main stocks: “West”, which includes individuals from Azores and Madeira (part of the Macaronesian region in the Atlantic), “Central”, which corresponds to Iberia (the center of the sampling distribution) and Northern Morocco, and “East”, which gathers the Mediterranean samples and those from the Canary Islands. The observed genetic differentiation between Mediterranean and Atlantic populations (except the Canary Islands) is in agreement with previous phenotypic and genetic studies based on mtDNA [18,23,26], suggesting the existence of a phylogeographic break between the South of Portugal and Mediterranean populations. The Almeria–Oran Front is likely mainly responsible for the reduction in gene flow among populations on each side, as previously observed in sardine [30] and other species [57,58]. In fact, the shared pattern of ancestry of the individuals from the Alboran Sea and the Gulf of Cadiz indicates that the Strait of Gibraltar is not such a strong barrier as previously suggested [27,29,30]. However, a small proportion of ancestry associated with the Central cluster can be observed across the Mediterranean, except for the Adriatic Sea (Figure 1B). The singularity of the sardine populations in the Northern Adriatic Sea has been suggested to result from a bottleneck linked to heavy fishing combined with oceanographic isolation [59]. The impact of gene flow between the Center and Mediterranean populations could also explain the sharing of mitochondrial haplotypes (Figure S1).

The Western group has also been observed with microsatellite data [10], which revealed high differentiation between Azores/Madeira and the other Atlantic populations. Notably, populations from these two archipelagos cluster together genetically, despite Madeira being geographically closer to Canary Islands and almost at the same distance to Iberia as it is to Azores. This strongly suggests a barrier to gene flow between the region formed by these two archipelagos and the other populations analyzed in this study, including Canary Islands and Iberia. This genetic division can be caused by currents, isolation by distance and a lack of suitable habitat between these regions, local adaptation to different environmental conditions, or other reasons. The fact that we did not observe a pattern consistent with isolation by distance and that we excluded markers putatively under selection argues against the latter. Nonetheless, this needs further investigation.

The higher differentiation of sardine populations from Azores and Madeira is also clear in the mitogenome network (Figure S1). Although two other main clades are observed, they are formed by haplotypes from individuals with a very different nuclear-based ancestry. Thus, it is not easy to objectively pinpoint the geographic origin of these mtDNA clades.

Discordance between differentially inherited markers can simply result from stochastic patterns of lineage sorting, but it can also be indicative of introgression [60]. Patterns suggesting admixture between the three genetic clusters were also observed with the nuclear data in all populations except Madeira. Given the lower effective population size of mtDNA when compared to nuclear DNA, we would expect to see it more sorted within each region.

An important piece of information that can help us to disentangle the role of gene flow versus shared ancestral polymorphism is the geographic pattern of differentiation. Genetic differentiation is lower between closer geographic populations within the East and Center clusters (Figure 3). Furthermore, we observed that the proportion of individuals with pure nuclear ancestry is higher in populations that are geographically more distant from populations with a different ancestry, suggesting that at least some of the patterns observed with nuclear and mtDNA genomes can indeed be created by gene flow between populations from these genetic clusters. Although this needs to be further confirmed using model-based approaches, if true, it provides additional support that the barriers involved in the differentiation between these three genetic clusters are only partial. Furthermore, the ancestry patterns observed between populations from the Central and Eastern clusters could suggest bidirectional gene flow between populations from Iberia and Mediterranean populations outside Iberia, which is also supported by the more even distribution of the two main mitochondrial haplotypes between these regions.

Western cluster ancestry is also observed in populations from the Center, Canary Islands and mainly in the Western Mediterranean populations. Although these patterns are compatible with admixture, gene flow between populations from the Eastern and Western clades is more difficult to explain. This discordance between molecular markers can also reflect the fact that regional populations of sardines seem to undergo periodic extinctions and recolonizations [61]. A recolonization of the Mediterranean from a refugium on the West African coast, as it has been suggested for anchovies [62], a species that shares several traits with sardines [63], could potentially explain the admixed ancestry of the Canary Islands and the Eastern cluster (Figure 3). The Canary Current upwelling system [64] could afterwards have been acting as a partial barrier to gene flow, maintaining the affiliation between the Canary Islands and the Mediterranean individuals. Some isolation between the archipelagos and the continent has also been found in other pelagic fish species using morphological traits [65,66,67].

**Figure 5:**
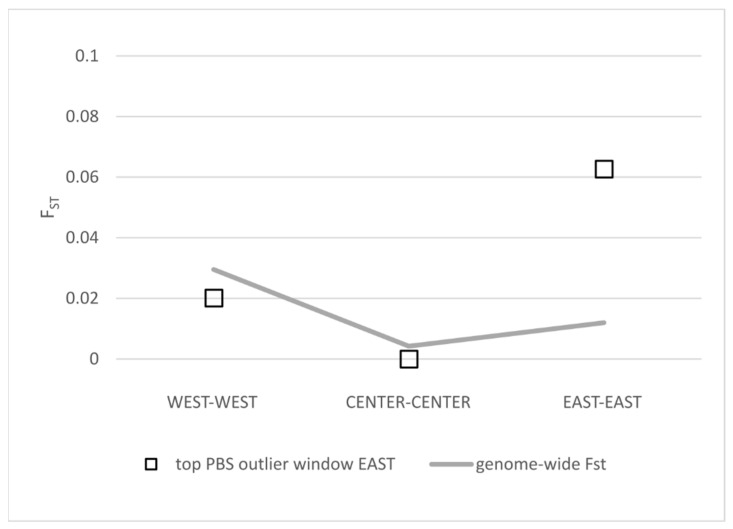
Comparison of F_ST_ values for the top PBS outlier window putatively associated with otolith development within three geographical regions: WEST (Azores vs. Madeira), CENTER (Bay of Biscay vs. Gulf of Cádiz) and EAST (Adriatic vs. Aegean).

Finally, we found that genomic regions corresponding to the top outliers of genetic differentiation are located in areas of low recombination (Figure 4), suggesting that genetic architecture can be contributing to some extent to the observed pattern of population structure. Interestingly, one of these regions includes genes related to otolith formation. Otolith shapes have been found to divide Atlantic and Mediterranean sardines [27] and support subdivisions within the Mediterranean populations [68]. We observed that the F_ST_ values for this genomic region are very high compared to the average genome-wide value when including the Aegean population (Figure S3). This is also valid when comparing with the Adriatic population (Figure 5), which could potentially contribute to the observed regional anatomical divergence within the Mediterranean [68]. Future work, including larger sampling sizes and associated phenotype information, is required to assess how variation in specific regions of the genome affects phenotypes of interest for fisheries and stock assessment.

## 5. Conclusions

Our main results provide evidence for three main genetic clusters of sardine populations across the analyzed specimens, suggesting at least two important barriers to gene flow. Although these do not seem complete, with gene flow possibly occurring between the three main phylogeographic regions identified, they seem to be strong enough to maintain populations genetically differentiated following their evolutionary trajectory. Our results thus offer an important baseline for further studies trying to identify the nature of these and other possible barriers between sardine populations, which can be compared with the phylogeographic patterns of other organisms with a similar distribution. Finally, the differentiation patterns reported here, together with the genetic resources generated for this commercially important species, offer information of strategic importance for transnational stock management of this highly exploited species towards sustainable fisheries.

## Figures and Tables

**Table 1:** Sampling information including our assignment to the three observed genetic clusters (Figure 1B).

Location	Short ID	Latitude	Longitude	Source	Collection Date (Number of Individuals)	Genetic Cluster	Tissue
Azores	AZO	37.8	−26.7	Fish market	June 2017 (10)	West	Muscle
Madeira	MAD	32.0	−16.9	Fish market	July 2017 (10)	West	Muscle
Canary Islands	CAN	28.8	−15.0	Collaborators	July 2017 (5), June 2022 (5)	East	Muscle
Bretagne	BRE	48.3	−4.9	Fish market	July 2017 (3)	Center	Muscle
Bay Biscay C	BCX	43.3	−1.9	[32]	December 2018 (5)	Center	Pectoral fin
Bay Biscay C	BC0	43.5	−1.7	PELACUS	Spring 2015 (5)	Center	Muscle
Bay Biscay S	BS0	43.8	−7.6	PELACUS	Spring 2015 (5)	Center	Fin
Iberia NW	IB1	42.2	−9.6	Fish market	June 2017 (5)	Center	Muscle
Iberia NW	IB2	41.1	−9.6	Fish market	June 2017 (5)	Center	Muscle
Gulf of Cádiz	CAD	37.0	−7.9	[32]	October 2018 (5)	Center	Pectoral fin
Morocco	MOR	34.5	−8.2	Collaborators	July 2017 (5)	Center	Muscle
Alboran Sea	ALB	36.1	−3.8	PELACUS	2017 (5)	Center	Muscle
Mar Menor	MME	38.0	−0.7	[32]	November 2018 (5)	East	Dorsal fin
Gulf of Lion	LIO	43.4	3.7	[32]	November 2018 (5)	East	Pectoral fin
Tunisia	TUN	37.7	10.8	Fish market	August 2017 (5)	East	Muscle
Adriatic Sea	ADR	44.9	13.1	Fish market	August 2017 (10)	East	Muscle
Aegean Sea	AEG	40.2	22.9	Fish market	August 2017 (10)	East	Muscle

## Data Availability

The Illumina fastq files raw reads are deposited in *NCBI Sequence Read Archive* (Bioproject accession no.: PRJNA688514, samples accession no.: SRR13325046, SRR13324980, SRR13324981, SRR13324982, SRR13324985, SRR13324986, SRR13324988, SRR13324989, SRR13324991, SRR13324992, SRR13324999, SRR13325000, SRR13325002, SRR13325003, SRR13325007, SRR13325008, SRR13325010, SRR13325011, SRR13325013, SRR13325014, SRR13325015, SRR13325018, SRR13325019, SRR13325021, SRR13325022, SRR13325023, SRR13325024, SRR13325025, SRR13325026, SRR13325027, SRR13325028, SRR13325029, SRR13325030, SRR13325031, SRR13325032, SRR13325033, SRR13325034, SRR13325035, SRR13325036, SRR13325037, SRR13325038, SRR13325039, SRR13325040, SRR13325041, SRR13325042, SRR13325043, SRR13325044, SRR13325045, SRR13325047, SRR13325048, SRR13325049, SRR13325050, SRR13325051, SRR13325052, SRR13325053, SRR13325054, SRR13325055, SRR13325056, SRR13325057, SRR13325058, SRR13325059, SRR13324977, SRR13324978, SRR13324979, SRR13324983, SRR13324984, SRR13324987, SRR13324990, SRR13324993, SRR13324994, SRR13324995, SRR13324996, SRR13324997, SRR13324998, SRR13325001, SRR13325005, SRR13325006, SRR13325004, SRR13325009, SRR13325012, SRR13325016, SRR13325017, SRR13325020, SRR13325060). The data will be public upon acceptance of the manuscript for publication.

## Supplementary Materials

The following supporting information can be downloaded at: https://www.mdpi.com/article/10.3390/genes15020170/s1.
