# Supplementary material for: Population Genomics Reveals the Underlying Structure of the Small Pelagic European Sardine and Suggests Low Connectivity within Macaronesia
Source: Genes (Basel). 2024 Jan 27;15(2):170. doi: 10.3390/genes15020170 (PMC10888339; doi:10.3390/genes15020170)
Supplement: Supplementary file 1 [file genes-15-00170-s001.zip › SupplementaryFigures.pdf]

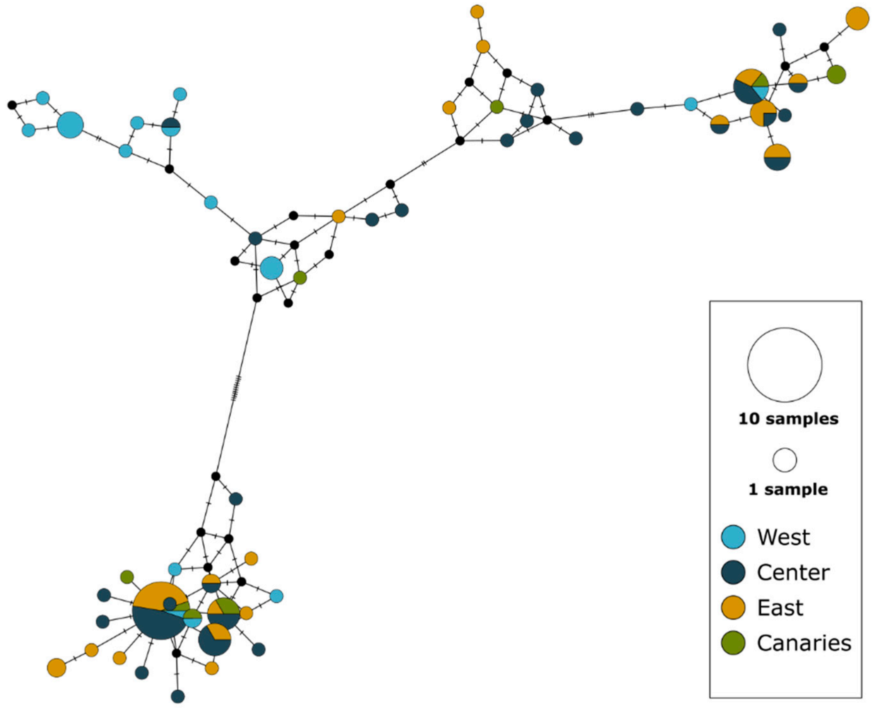

Figure S1. Population net (PopART, median-joining network) obtained using mitochondrial variants with minor allele frequency above 30%; mutations shown as Hatch marks. Colors represent the main ancestry of each individual (for K=3 as in Figure 1B) except for samples from the Canary Islands which are depicted in green.

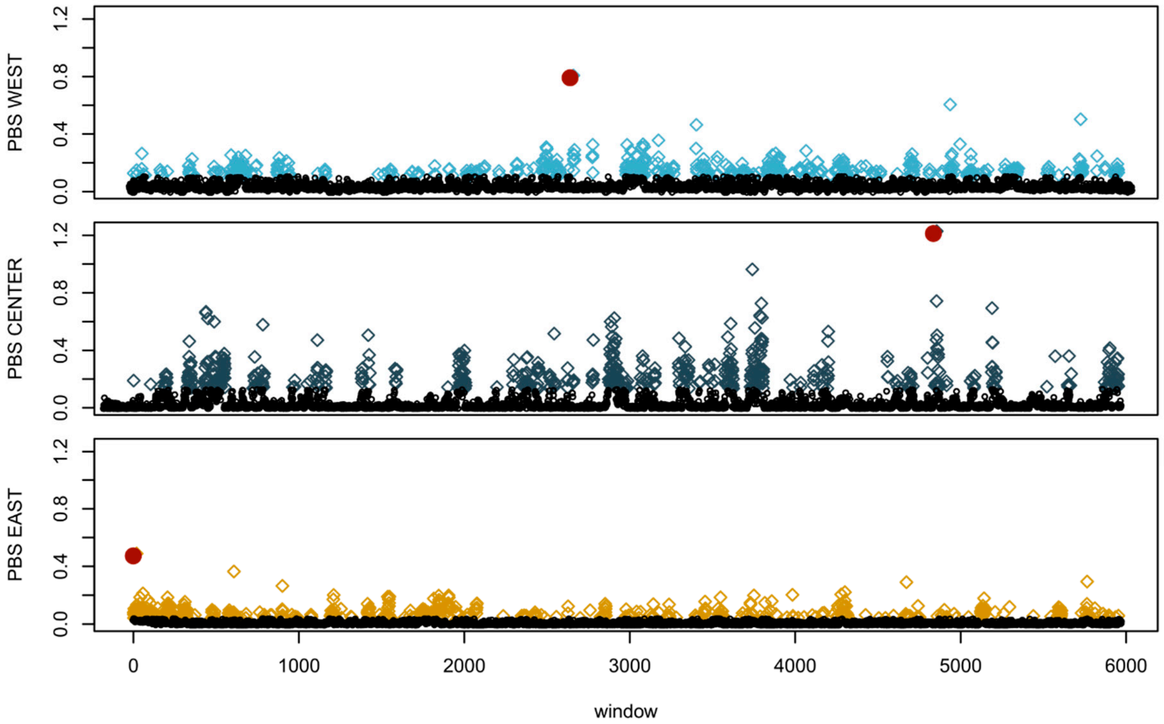

Figure S2. Distribution of the PBS values. Genomic regions (50 kb windows) with PBS values below the 90th percentile (putatively neutral) are shown in black. Red dots show the top outlier.

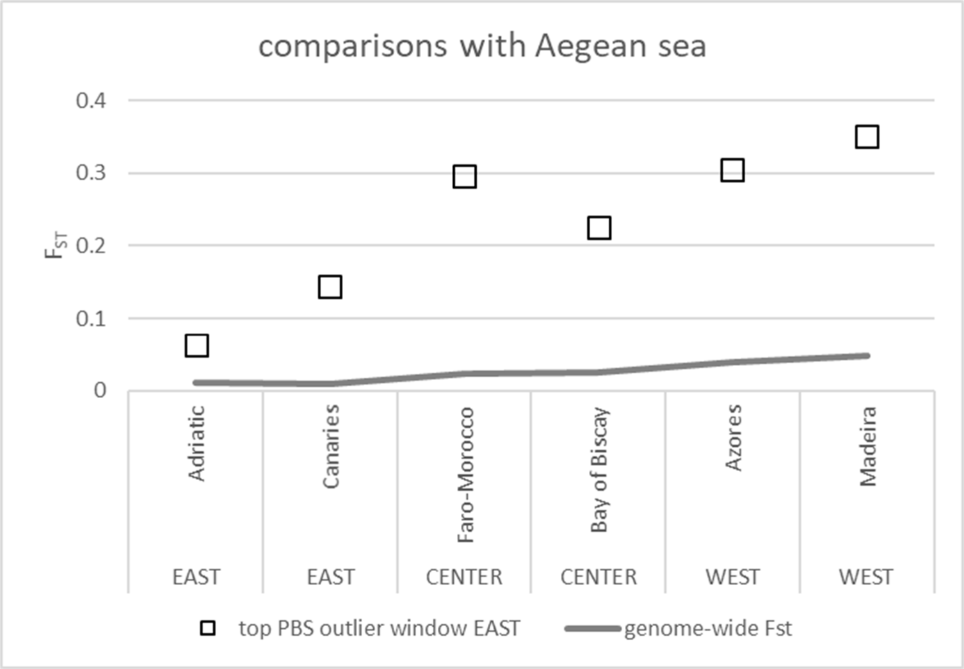

Figure S3. Comparison of F_{ST} values for the top PBS outlier window putatively associated with otolith development vs the genome-wide average F_{ST}.
